# Rosinidin Protects Streptozotocin-Induced Memory Impairment-Activated Neurotoxicity by Suppressing Oxidative Stress and Inflammatory Mediators in Rats

**DOI:** 10.3390/medicina58080993

**Published:** 2022-07-26

**Authors:** Khalid Saad Alharbi, Muhammad Afzal, Sami I. Alzarea, Shah Alam Khan, Fadhel A. Alomar, Imran Kazmi

**Affiliations:** 1 Department of Pharmacology, College of Pharmacy, Jouf University, Sakaka 72341, Saudi Arabia; kssalharbi@ju.edu.sa (K.S.A.); samisz@ju.edu.sa (S.I.A.); 2 Department of Pharmaceutical Chemistry, College of Pharmacy, National University of Science and Technology, Muscat 130, Oman; shahalam@nu.edu.om; 3 Department of Pharmacology and Toxicology, College of Clinical Pharmacy, Imam Abdulrahman Bin Faisal University, P.O. Box 1982, Dammam 31441, Saudi Arabia; falomar@iau.edu.sa; 4 Department of Biochemistry, Faculty of Science, King Abdulaziz University, Jeddah 21589, Saudi Arabia

**Keywords:** rosinidin, streptozotocin, neurotoxicity, oxidative stress

## Abstract

*Background and Objectives*: To assess the antioxidant and neuroprotective role of rosinidin on rat memory impairment that is induced by streptozotocin. *Materials and Methods*: Wistar rats were given an intraperitoneal (i.p) injection of streptozotocin (60 mg/kg) followed by treatment with rosinidin at selective doses (10 and 20 mg/kg) for 30 days. The behavioral parameters were estimated by Y-maze test and Morris water test. Biochemical parameters such as acetylcholinesterase (AChE), choline aacetyltransferase (ChAT), and nitric oxide, and antioxidants such as glutathione transferase (GSH), superoxide dismutase (SOD) IL-6, IL-10, Nrf2, and BDNF, were determined. *Results*: The study results revealed that rosinidin improved cognition by reverting the behavioral parameters. The treatment with rosinidin restored the antioxidant enzymes and inflammatory cytokines. *Conclusions*: From the results, it has been proven that rosinidin possesses antioxidant, anti-amnesic, and anti-inflammatory activity. Rosinidin improved the cognitive and behavioral deficits that were induced by streptozotocin. Furthermore, 20 mg/kg rosinidin was found to have strong protective action against streptozotocin-induced toxicity.

## 1. Introduction

Neurodegenerative disorders, such as Alzheimer’s disease, Parkinson’s disease, and Huntington’s disease, are the most serious conditions that are linked with neuronal damage that is caused by oxidative stress [1,2]. Neurodegeneration leads to cognitive impairment, which is caused by an imbalance in neurotransmitter release, oxidative damage, and genetic dysfunction [3,4]. Oxidative stress is caused by insufficiency in the antioxidant enzymes and reactive oxygen species (ROS), which results in excess ROS generation that slows down the function of biomolecules leading to neuronal damage [5,6]. Oxidative stress majorly affects the cholinergic neurons, causing a reduction in the AchE level by decreasing the choline acetyltransferase enzyme which regulates acetylcholine activity [7]. Oxidative stress also triggers the hypothalamic-pituitary axis (HPA) to release glucocorticoids, which causes shrinkage of the brain and an impairment of cognitive functions [8,9].

Streptozotocin is a nitrosourea derivative belonging to the antibiotic class. It is mostly used to induce diabetes by targeting the β-pancreatic cells in experimental animals [10]. When intraperitoneally injected, streptozotocin produces oxidative stress in rats, causing neuronal damage [11]. It impairs brain function, glucose metabolism in the brain, cholinergic neurons, and causes rise in free radical generation, leading to memory and cognitive dysfunction [12,13]. Oxidative damage that is produced by streptozotocin is implicated in several neurodegenerative disorders. Treating it with antioxidants would aid in managing the oxidative damage that is produced in neuro-disorders [14]. Streptozotocin increases the inflammatory, oxidative stress markers, NFKB, and Nrf-2 [15,16]. 

Flavonoids have a wide acceptance in managing neurodegenerative conditions disease, such as Parkinson’s and Alzheimer’s, by activating antioxidative markers, hindering inflammatory mediators, and exhibiting neuroprotective action in several animal models [16,17]. Anthocyanins and anthocyanidins, a flavonoid, have a potential role in neurodegenerative diseases [18]. Anthocyanins have been reported to increase the neurotransmitter and brain-derived neurotrophic factor (BDNF) levels in depressive rodents [19].

Rosinidin, an anthocyanidin, is mainly found in plants, namely *Primula Rosea* and *Catharanthus roseus* [20]. Chemically, rosinidin contains a benzopyrylium ring with hydroxyl groups that are attached to 3′ and 4′ positions. In silico studies showed that rosinidin possesses certain pharmacological actions, such as antioxidant and anti-inflammatory, which could be beneficial in treating neurodegenerative disorders [21]. Docking studies also proved the neuroprotective action of rosinidin in Parkinson’s disease. Hence, from the above study, we aim to assess the effect of rosinidin in streptozotocin-activated memory impairment-activated neurotoxicity.

## 2. Materials and Methods

### 2.1. Chemicals

Streptozotocin was obtained from Sigma (St. Louis, MI, USA). The commercially available (Modern Lab, Maharashtra, India) biochemical estimation kits for IL-6, IL-1β, and TNF-α were used in the study. Other reagents and chemicals were acquired from local sources (Modern Lab, Maharashtra, India) and were of standard quality.

### 2.2. Animals 

Adult male Wistar rats (150–200 g) were segregated in propylene cages and maintained at room temperature (24 ± 5 °C), with a relative humidity (50–60%), standard pellet food, and a surplus amount of water under 12 hr dark and light cycles. The study was approved by the Institutional Animal Ethics Committee (IAEC-TRS/PT/021/005).

### 2.3. Experimental Protocol

A total of four groups with six rats in each group were randomized after acclimatizing for a week.

Group I—Normal
Group II—Streptozotocin + Saline
Group III—Streptozotocin + Rosinidin-10 mg/kg
Group IV—Streptozotocin + Rosinidin-20 mg/kg

**Figure 1:**
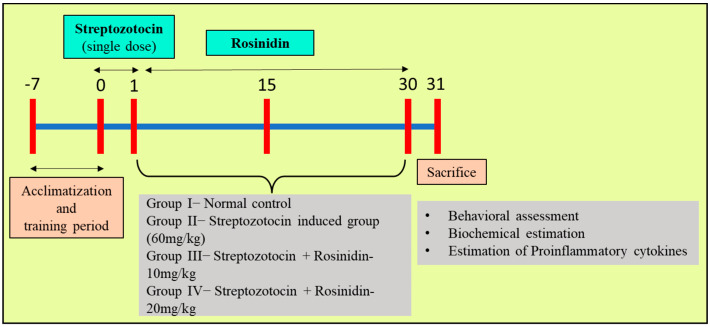
Experimental plan.

The normal group received the vehicle (2 mL/kg) for 30 days. The streptozotocin group received a single dose of streptozotocin (60 mg/kg) injection intraperitoneally (i.p.) and saline for the subsequent 30 days [22,23]. The treatment groups received dosing of the test drug (rosinidin) an hour after streptozotocin administration, starting from day 0 until 30 days (Figure 1). After the treatment period, all the animals were subjected to behavioral assessment, i.e., Y-maze test and then Morris water maze test. Post-behavioral-tested rats were sacrificed, and the brains were isolated and stored to perform biochemical estimations. All efforts were made to cause minimal distress to the experimental animals during the study. 

### 2.4. Acute Toxicological Studies

The study followed OECD Guideline Acute Toxicity Study No. 423 [24,25]. The test drug was orally administered to the rats at the maximum dose. The animals were observed for the next 14 days for remarked toxicity. Clinical patterns including behavioral changes and changes in the skin, fur, eyes, and body weight were noted. 

### 2.5. Motor Function

#### 2.5.1. Y-Maze Test

The Y-maze includes three arms: A, B, and C, angled at 120°, and covered with a black acryl. The rats were trained by placing the animal at the intersection of the three arms. The rats were replaced and again positioned at the center and were permitted to roam around in all of the arms. After the training period, the animals were dosed with the treatment drug and the same test was repeated. The total entries into the arms were noted when all the paws were on the floor of arms. Successive entries into each arm were measured when the rat enters the same arm in an alternate order. The total entries were also measured to check the cognitive performance of the disease control and treated rats [26,27].

#### 2.5.2. Morris Water Maze Test

This behavioral test is to assess the spatial learning and cognition of rats. The rats were trained for at least five days before performing the probe test. It consists of a water-filled circular tank (150 cm) with a raised platform (10 cm) 1 cm down the water surface. The animals were made to swim across in order to climb out the platform in 90 seconds. During the training session, a visible platform trial was conducted by attaching a flag (5–6 cm) to the platform so the animals could find the platform easily. The probe test was performed after rosinidin treatment. After which, the invisible trial was performed, where the animals were allowed to reach out to the platform without any sign. After the training period, the probe trial was performed without replacing the platform. The time taken in the area where the platform was placed was recorded [28,29].

### 2.6. Biochemical Parameters

Sacrifice and Homogenization

On the 29th day, after the behavioral analysis, the rats were subjected to biochemical estimation and determination of inflammatory cytokines. The isolated brain was homogenized using phosphate buffer and centrifugated for 15 min at 2000–3000 rpm. The supernatant was separated and reserved at 4 °C for biochemical assessment.

#### 2.6.1. Acetylcholinesterase (AchE) and Choline Acetyltransferase (ChAT)

The quantitative assay for AchE was performed by following the Ellman et al. method (1961). To 0.5 mL supernatant, 3 mL of phosphate buffer (0.01 mol/L), 0.1 mL acetylthiocholine iodide, and 0.1 mL Ellman’s reagent were mixed. The resultant reaction mixture was determined spectrometrically at 412 nm. The AchE activity was estimated in nmol/mg protein [30]. 

ChAT was estimated by a radiometric method as described below. The reaction mixture contains acetyl-CoA, acetyl-CoA with SPA, NaCl, MgCl_2_, physostigmine salicylate, choline chloride, Triton, albumin, sodium phosphate buffer, EDTA, and the sample. The mixture was incubated for 40–50 mins at 45 °C, then cooled. Ach was then added to the buffer. After which, sodium tetraphenylborate was added and the organic phase was separated and measured by beta counter [31]. 

#### 2.6.2. Antioxidant Enzyme Estimation

##### Malondialdehyde Determination (MDA)

Malondialdehyde is the end-product that is formed after lipid peroxidation. The mixture involves an equal volume of supernatant and trichloroacetic acid (10% *w/v*) thoroughly mixed, cooled in ice water, and centrifugated at 3000 rpm. To the collected supernatant, thiobarbituric acid was added and heated for 15 mins to complete the reaction process. The absorbance was recorded at 535 nm, spectrometrically. The quantity of MDA was represented as nM of MDA/g of wet tissue [32]. 

##### Reduced Glutathione (GSH) Estimation

To the homogenate, trichloroacetic acid (10% *w/v*) was mixed. The collected supernatant was reacted with phosphate buffer (0.3 M) and 0.5 mL DTNB reagent (5-5′-Dithio-Bis (2 Nitro-benzoic acid)). The mixture was spectroscopically recorded at 412 nm and was expressed as nmol GSH/gm wet tissue [33].

##### Superoxide Dismutase (SOD)

The reaction mixture was composed of Tris buffer (50 mM), EDTA (1 Mm), enzyme supernatant (100 µL), pyrogallol (0.4 mM), and distilled water. The reaction mixture was measured for 3 min by UV spectrophotometer at 420 nm. The inhibition of pyrogallol was due to the presence of superoxide dismutase, which was indirectly recorded. One unit is calculated as the amount of SOD that is required to inhibit 50% pyrogallol under standard assay conditions and is presented as SOD units/mg protein [34,35].

##### Catalase (CAT) Assay

The activity of catalase depends upon the rate of H_2_O_2_ decomposition to water and molecular oxygen. The assay procedure consists 0.5 mL homogenate and 30 mM H_2_O_2_ (freshly prepared) mixed in 0.05 M phosphate buffer. The mixture is spectroscopically analyzed at 212 nm. The catalase activity was represented as units of catalase/wet gm of tissue [36].

#### 2.6.3. Nitrite Estimation 

The nitric oxide that was produced in the supernatant was estimated by colorimetric assay given by Green et al. (1982). The reaction mixture contains an equal amount of supernatant and Griess reagent (Naphthyl ethylene diamine and sulphanilamide in H_3_PO_4_). After 10-min incubation, the change in the absorbance was estimated at 540 nm spectrometrically. The nitrite concentration was determined by standard curve and shown as µmol/mg protein [37].

#### 2.6.4. Neuroinflammatory Parameters

Proinflammatory cytokines such as TNF-α, IL-1β, IL-6, IL-10, NF-kb, Nrf2, and BDNF protein expression were estimated by commercially available immunoassay (ELISA) kits. Nrf2 activity was measured using the NEPER kit in the nuclear fraction by following the instructions. The concentration was expressed as pg/mL protein. The protein level was assessed by Bradford’s test [38]. 

### 2.7. Statistical Analysis

With the exception of the Morris’ water maze, which was analyzed with two-way ANOVA followed by Bonferroni’s test using GraphPad prism, the results of each parameter were analyzed by one-way ANOVA followed by Tukey’s post hoc comparison. Data significance was determined at *p* < 0.05.

## 3. Results

### 3.1. Acute Toxicity Study

During the 14-days of the acute toxicity study, no morbidity or clinical appearance of symptoms were noticed. Hence, based on the acute oral toxicity studies, we decided to conduct the main investigation with 10 mg/kg and 20 mg/kg rosinidin for performing the main study.

### 3.2. Behavioural Tests

**Y-maze Test**

**Figure 2:**
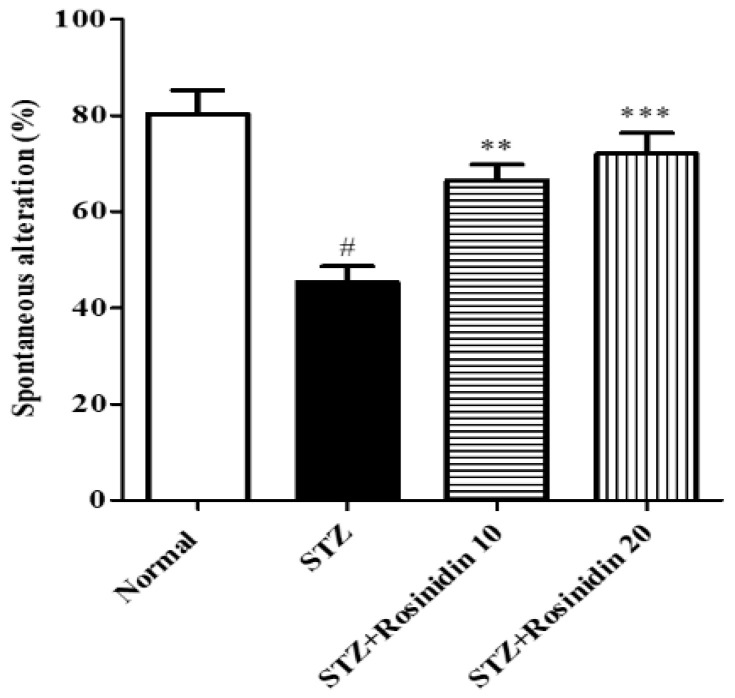
The effect of rosinidin on the Y-maze test. All the values are presented as the mean ± SEM. Correlation among the groups was done using Tukey’s test by one-way ANOVA. *p* value < 0.01, 0.001 were expressed as **, *** respectively as compared with the streptozotocin (STZ) group. *p* < 0.001 were expressed as # when compared with the controls.

This test was performed to check the working memory function of streptozotocin-induced rats after treatment with rosinidin. The streptozotocin-induced rats showed a marked decline (*p* < 0.001, 43.41%) in the % spontaneous alterations as compared to the controls while the rosinidin-treated rats demonstrated a rise in 10 mg/kg (*p* < 0.01, 17.28%) and 20 mg/kg (*p* < 0.001, 10.28%) in the activity as compared with the streptozotocin-induced group. The total number of entries into each arm by the streptozotocin-induced group decreased (*p* < 0.001) compared with the blank group. However, treatment with rosinidin showed an increase in number of entries as compared with the streptozotocin group (Figure 2).

**Morris Water Maze**

**Figure 3:**
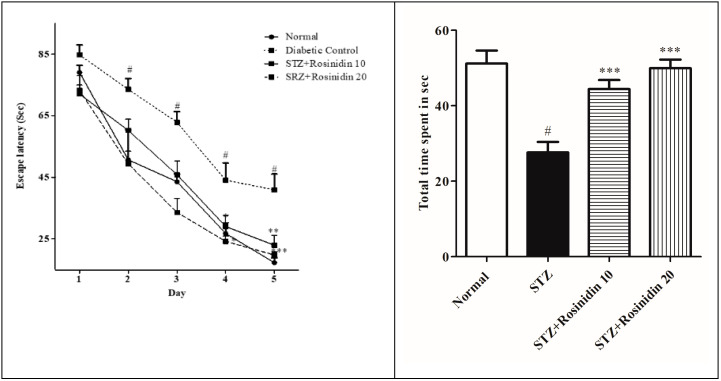
The effect of rosinidin on the Morris water maze test. All the values are presented as the mean ± SEM. Correlation among the groups was done using two-way ANOVA. *p* value < 0.01, 0.001 were expressed as **, *** respectively as compared to the streptozotocin (STZ) group. *p* < 0.001 were expressed as # when compared with the controls.

To check the spatial memory of the rosinidin-treated group, the rats were subjected to a maze test. During the training days, all the groups displayed a decline in the escape latency. The streptozotocin-induced group had a higher (*p* < 0.001) escape latency time from the second day as compared with the control groups. While from Day 2, the rosinidin-treated group dose-dependently reduced (*p* < 0.01 and *p* < 0.001) the latency time when compared with the streptozotocin group. In the probe trial, one-way ANOVA revealed that streptozotocin-induced (46.60%) rats spent less time in the target quadrant as compared to the rats that were treated with rosinidin. Furthermore, post hoc analysis revealed that rosinidin at both doses (10 mg/kg, 13.83%; 20 mg/kg, 2.38%) significantly increased the time that was spent in the target quadrant compared to streptozotocin control rats (*p* < 0.05) (Figure 3). 

### 3.3. Biochemical Parameters

**Acetylcholinesterase and Choline Acetyltransferase**

**Figure 4:**
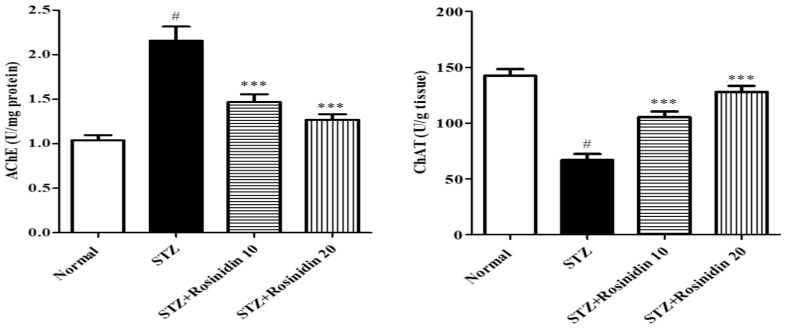
The effect of rosinidin on AChE and ChAT activity. All the values are presented as the mean ± SEM. Correlation among the groups was done using Tukey’s test by one-way ANOVA. *p*-value < 0.001 were expressed as *** respectively as compared with the streptozotocin (STZ) group. # significant as compared with the controls (*p* < 0.001).

The streptozotocin-induced group displayed a remarkable rise (*p* < 0.001, −108.1%) in the AchE level compared with the controls. When treated with rosinidin, both doses showed a marked decrease (*p* < 0.001, 10 mg/kg, −41.75%; 20 mg, −22.46%) in the AchE level as compared to the streptozotocin group, suggesting less breakdown of acetylcholine (Figure 4). 

A sharp reduction (*p* < 0.001, 52.98%) in the ChAT level was observed in the streptozotocin-induced rats compared to the normal group. While rosinidin at both doses markedly elevated (*p* < 0.001, 26.03; 10.17%) the ChAT levels when compared to the streptozotocin-induced rats.

**Antioxidant Parameters**

**Figure 5:**
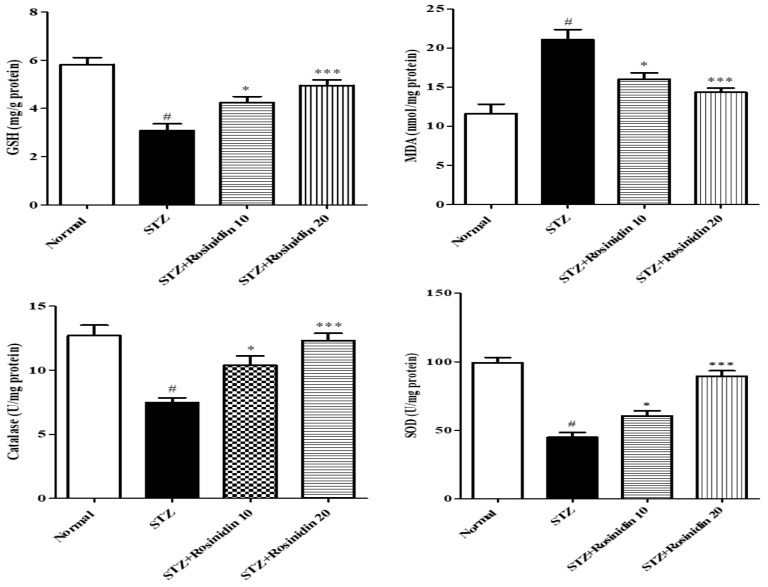
The effect of rosinidin on antioxidant enzyme activities. All the values are presented as the mean ± SEM. Correlation among the groups was done using Tukey’s test by one-way ANOVA. *p*-value < 0.001, 0.05 were expressed as ***, * respectively as compared with the streptozotocin (STZ) group. # significant as compared with the controls (*p* < 0.001).

The MDA level in the streptozotocin group increased (*p* < 0.001, −81.89%) significantly as compared with the controls. Treatment with 10 mg/kg (*p* < 0.05, −38.18%) and 20 mg/kg (*p* < 0.001, −23.70%) rosinidin decreased the lipid peroxidation (MDA) in comparison to the streptozotocin group. A marked downfall (*p* < 0.001, 47.02%; 54.77%; 41.20%) in GSH, SOD, and CAT was recorded in the streptozotocin groups compared to the normal group. With 10 mg/kg (*p* < 0.05, 27.18; 39.18%; 18.11%) and 20 mg/kg (*p* < 0.001, 16.63%;9.93%; 2.99%) rosinidin, a noticeable increase in the GSH, SOD, and CAT level was noticed when compared with the streptozotocin group (Figure 5).

**Nitrite Assay**

**Figure 6:**
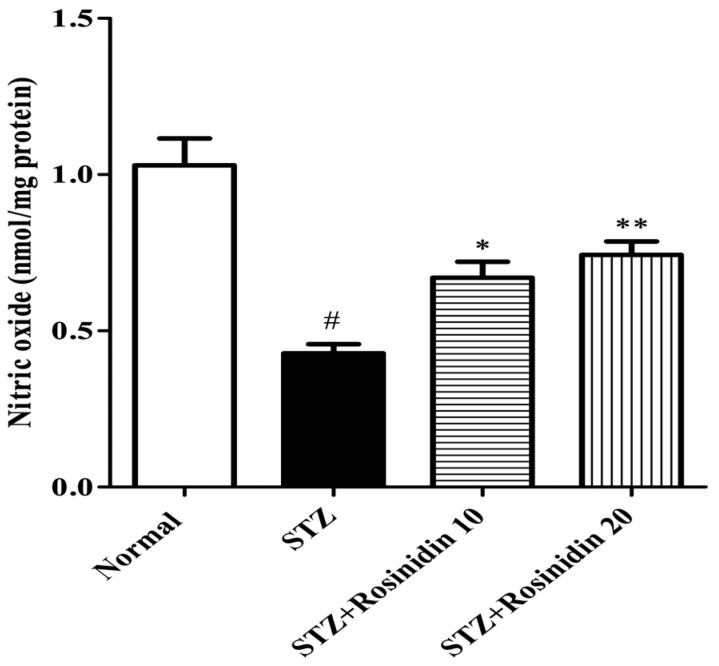
The effect of rosinidin on nitric oxide level. All the values are presented as the mean ± SEM. Correlation among the groups was done using Tukey’s test by one-way ANOVA. *p*-value < 0.05, 0.01 were expressed as *, ** respectively as compared with the streptozotocin (STZ) group. # significant as compared with the controls (*p* < 0.001).

The streptozotocin group displayed a sharp decline (*p* < 0.001, 58.41%) in the nitric oxide level when compared with the control group. On the other hand, for the treatment groups, 10 mg/kg (*p* < 0.05, 34.95%) and 20 mg/kg (*p* < 0.01, 27.83%) rosinidin markedly enhanced the nitric oxide level when compared with the streptozotocin group (Figure 6).

**Neuroinflammatory Parameters**

The level of inflammatory markers (IL-1β, IL-6, TNF-α, NF-kB) in the streptozotocin group increased (*p* < 0.001, −121.64%; −154.85; −130.14%; −154.45%) significantly when compared with the control group. On the other hand, rosinidin at low dose (10 mg/kg) showed a less significant decrease (*p* < 0.05, −87.70; −115.98%; −100.06%; −59.82%) in the IL-1β, TNF-α, NF-kB, and IL-6 than the higher dose (20 mg/kg) (*p* < 0.001; −33.88%; −34.91%; −40.62%; −41.17%) in comparison to the streptozotocin group.

**Figure 7:**
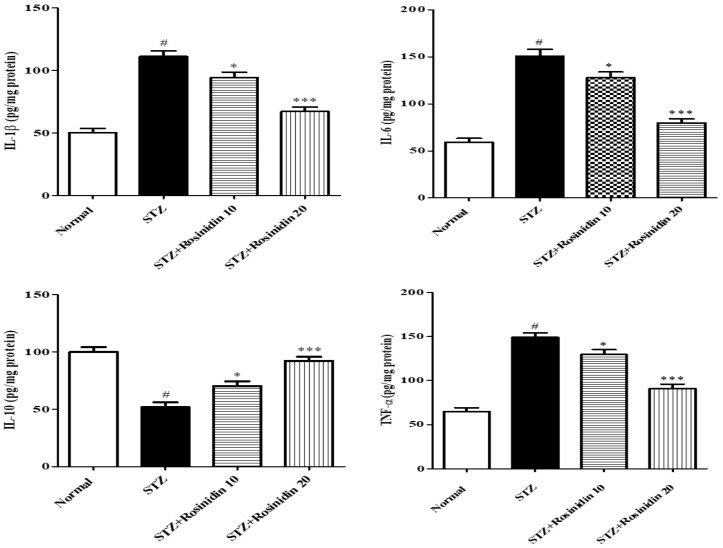
The effect of rosinidin on the neuroinflammatory cytokine levels. All the values are presented as the mean ± SEM. Correlation among the groups was done using Tukey’s test by one-way ANOVA. *p*-value < 0.001, 0.05 were expressed as ***, * respectively as compared with the streptozotocin (STZ) group. # significant as compared with the controls (*p* < 0.001).

**Figure 8:**
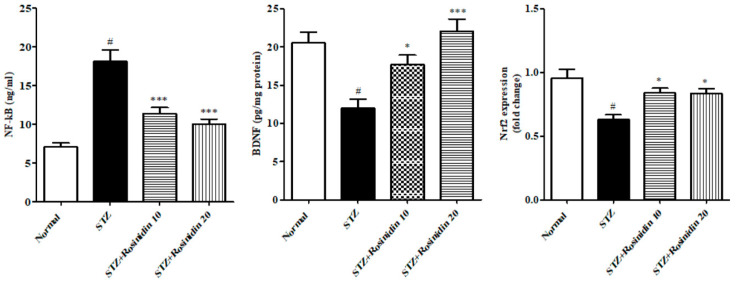
The effect of rosinidin on NF-kB, Nfr2, and BDNF proteins. All the values are presented as the mean ± SEM. Correlation among the groups was done using Tukey’s test by one-way ANOVA. *p*-value < 0.05, 0.001 were expressed as *, *** respectively as compared with the streptozotocin (STZ) group. # significant as compared with the controls (*p* < 0.001).

The IL-10, Nrf2, and BDNF protein levels decreased (*p* < 0.001, 48.24%; 34.03%; 41.54%) in the streptozotocin group as compared with the normal group. When treated with low dose (10 mg/kg) rosinidin, it displayed a rise in the IL-10, Nrf2, and BDNF (*p* < 0.05, 29.71%; 12.04%; 13.68%). When treated with a higher dose (20 mg/kg) rosinidin, a significant rise was observed in IL-10 (*p* < 0.001, 7.67%), Nrf2 (*p* < 0.05, 12.56%), and BDNF (*p* < 0.001, −7.50%) protein expression as compared with the streptozotocin group (Figure 7 and Figure 8).

## 4. Discussion

In the current study, acute toxicity of rosinidin was performed to assess the dose toxicity in rats. In an acute toxicity study, rats that were treated with 10 and 20 mg/kg rosinidin did not show any signs and symptoms of toxicity.

The present study showed that streptozotocin causes memory impairment and loss of motor coordination. The administration of streptozotocin injection desensitizes the insulin receptor that is present in the neurons, decreases the energy metabolism of the brain, and also hinders cholinergic transmission [39,40,41]. We carried out a Y-maze behavioral test to assess the spatial memory of the rats. The spontaneous activity was measured by observing the rats that enter the arm that they have previously visited. From the results, a significant decline in spontaneous alteration was noted in the streptozotocin group when compared to the control, which indicated behavioral toxicity that was the same as previously reported [42,43]. In the streptozotocin-treated group, the time and travelled paths were enhanced which are in agreement with similar studies of this type, such as the learning deficits with the severe impairment of Morris water maze performance in STZ-induced diabetes [41,44,45]. Treatment with rosinidin increased the % spontaneous alteration, thereby improving cognition. An alternative test, the Morris water maze test, was also performed to check the learning ability and cognition in diseased rats. Similar to previous studies, the results displayed a downfall in the escape latency and total entries in the streptozotocin group as compared with the controls [46,47]. Rosinidin treatment in the streptozotocin-induced rats dose-dependently enhanced memory acquisition and spatial learning ability, suggesting rosinidin can modify the brain functionality that has been slowed down by streptozotocin. 

The cholinergic system has a central role in regulating sensory functions, including cognition, arousal, and sleep patterns [48]. An alteration in the cholinergic system can cause a loss of memory and behavioral disturbances. Streptozotocin administration leads to a deficiency of cholinergic neurons, which is followed by a decline in the ChAT enzyme and a rise in AChE as previously reported [49,50,51]. Rosinidin treatment markedly decreased the AchE activity and improved the cognitive dysfunctions in streptozotocin-activated neurotoxicity. It proved that rosinidin possesses AchE inhibitory activity, which is due to the antioxidant potentiality that elevated the oxidative defense mechanism of the cholinergic neurons and thus ameliorated behavioral and cognitive deficits. 

Brain cells are the highest producer of oxygen and are more prone to the attack of free radicals that are generated by oxidative stress, resulting in the decrease of antioxidant enzymes [52]. The neuronal cell membranes are usually composed of polyunsaturated fatty acids, which are also prone to lipid peroxidation [53]. In the current study, streptozotocin causes a rise in the lipid peroxidative damage and a downfall in antioxidant enzymes including GSH, SOD, CAT, and nitric oxide levels, indicating higher nitroso-oxidative stress, which is similar to the earlier studies [54,55,56]. Rosinidin dose-dependently reduced streptozotocin-activated oxidative damage and restored the antioxidant level close to normal, indicating its antioxidant action. The downfall in the nitric oxide by treating with rosinidin might be due to inhibition in the iNOS transcription and a decrease in the NOS activity. 

The produced reactive oxygen species have a major role in the activation of the NF-kB pathway, causing the overproduction of inflammatory cytokines. Similar to the previous studies, the present investigation results show that streptozotocin activates microglial cells, which produce inflammatory mediators and free radicals that damage neurons; increases inflammatory cytokines including TNF-α, IL-1β, IL-6, and NF-kB; and decreases IL-10, Nrf2, and BDNF protein expression [41,57,58,59]. Rosinidin dose-dependently reduced streptozotocin-induced inflammatory burden, proving anti-inflammatory action. Thus, from the pharmacological screening, it was found that rosinidin possesses anti-oxidant, anti-amnesic, and anti-inflammatory actions, indicating neuroprotection.

## 5. Conclusions

In the present investigation, rosinidin improved cognitive and spatial learning behavior in streptozotocin-induced rats, depicting anti-amnesic action. A reduction in AChE and restoration of antioxidant enzymes indicate the anti-oxidant mechanism of rosinidin against streptozotocin-induced neurotoxicity. Regulating the neuroinflammatory cytokines depicts the anti-inflammatory action of rosinidin in streptozotocin-induced memory impairment. Furthermore, the higher dose (20 mg/kg rosinidin) group showed greater neuroprotective activity than the low dose group. From the results, rosinidin can be chosen as an active natural constituent for further neurodegenerative disorder-related studies. 

## Data Availability

Not applicable.
